# Antarctica’s Protected Areas Are Inadequate, Unrepresentative, and at Risk

**DOI:** 10.1371/journal.pbio.1001888

**Published:** 2014-06-17

**Authors:** Justine D. Shaw, Aleks Terauds, Martin J. Riddle, Hugh P. Possingham, Steven L. Chown

**Affiliations:** 1 School of Biological Sciences, The University of Queensland, St. Lucia, Queensland, Australia; 2 Terrestrial and Nearshore Ecosystems, Australian Antarctic Division, Department of the Environment, Kingston, Tasmania, Australia; 3 School of Biological Sciences, Monash University, Clayton, Victoria, Australia

## Abstract

Antarctica is widely regarded as one of the planet's last true wildernesses, insulated from threat by its remoteness and declaration as a natural reserve dedicated to peace and science. However, rapidly growing human activity is accelerating threats to biodiversity. We determined how well the existing protected-area system represents terrestrial biodiversity and assessed the risk to protected areas from biological invasions, the region's most significant conservation threat. We found that Antarctica is one of the planet's least protected regions, with only 1.5% of its ice-free area formally designated as specially protected areas. Five of the distinct ice-free ecoregions have no specially designated areas for the protection of biodiversity. Every one of the 55 designated areas that protect Antarctica's biodiversity lies closer to sites of high human activity than expected by chance, and seven lie in high-risk areas for biological invasions. By any measure, including Aichi Target 11 under the Convention on Biological Diversity, Antarctic biodiversity is poorly protected by reserves, and those reserves are threatened.

## Abstract 2

Global comparisons show that Antarctica's terrestrial biodiversity is poorly protected. Existing protected areas are inadequate, unrepresentative, and threatened by increasing human activity.

## Introduction

With no permanent human settlements and science and tourism as the only land-based industries, Antarctica is under lower direct human pressure than any other continent. Its landscapes are recognized globally as the most pristine [1],[2] and can rightly be regarded part of the “last of the wild” [3]. Under the Antarctic Treaty System (ATS), through which the region is governed [4], the whole continent has been designated a “*natural reserve, devoted to peace and science*” [5]. Annex V of the Protocol on Environmental Protection to the Antarctic Treaty (Madrid Protocol) also makes provision for special protection of areas. Currently, 73 such Antarctic Specially Protected Areas (ASPAs) have been designated under nine categories (Table S1) representing their cultural, physical, or ecological values. Consequently, it is widely believed that the terrestrial Antarctic is more than adequately protected [6],[7]. In this perspective, we provide evidence that shows this is not the case.

## Why Protect Terrestrial Antarctica?

The majority of Antarctica's biodiversity is concentrated in ice-free areas. While there is life outside of these areas [8],[9], biodiversity and human activity are much higher within them. For these reasons, we focus on the protection of ice-free environments. Threats to the ecological integrity of Antarctica are accelerating because of a growing variety, intensity, and frequency of human activities and rapidly changing climates or forecasts for such change [10]–[12]. Biological invasions are most significant, with several established populations already having a variety of observed impacts [13]–[15]. Human activities in Antarctica typically take two forms: the activities of National Antarctic Programs (i.e., scientists and their support personnel) and those that take place as part of fee-paying recreation (i.e., tourists and their support personnel). Activities associated with science include construction of buildings, roads, fuel depots, and runways. Growing instances of unintentional damage are also being recorded, such as the establishment of harmful nonindigenous species, sewage spills, point source pollution, and destruction of vegetation [13],[16]–[19]. All human activities, be they tourism- or science-related, have increased considerably over the last 20 years and are predicted to continue to do so [20].

Protected areas are generally effective in reducing threats to biodiversity and regarded as the cornerstone of conservation [21]. Their efficacy is enhanced substantially when they are representative of the biodiversity of a region [22]. This is recognised by the Strategic Plan for Biodiversity 2011–2020, which has set 17% of terrestrial land area protected as the most recent target (Aichi Target 11) [23]. The Madrid Protocol likewise recognizes the significance of representative protected areas, calling for ASPAs to be identified *“within a systematic environmental-geographic framework*” and include “*representative examples of major…ecosystems*” [24].

Given that conservation threats to terrestrial Antarctica are growing, that protected areas are a cornerstone of conservation, and that a network of specially protected areas is already considered a key tool in the conservation management of Antarctica, we assess the effectiveness of this network in a contemporary global context. How representative is this network and, compared with other areas of Antarctica, how high are the risks to the network of nonindigenous species invasion, which at present pose the greatest conservation threat to the region [2],[25]?

Our assessment was conducted in three steps. We quantified the proportion of ice-free land that is protected, as this is where the majority of Antarctic biodiversity occurs; examined its representativeness using recently developed protected-area assessment metrics [26]; and quantified the level of threat these protected areas face from biological invasion using information from a recent, spatially explicit risk assessment (see Methods S1 for details of data sources and analytical methods) [25].

## Antarctic Protection Relative to Other Continents

**Figure 1:**
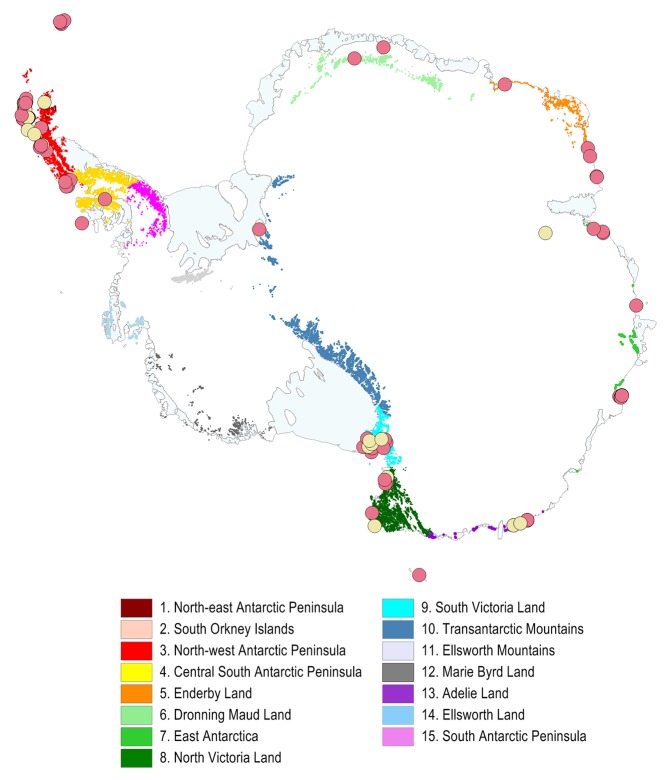
Location of Antarctic Specially Protected Areas (ASPAs) and Antarctic Conservation Biogeographic Regions (ACBRs). Red circles indicate ice-free ASPAs that protect terrestrial biodiversity, and yellow circles are ASPAs that are not ice-free or do not support terrestrial biodiversity, and therefore were not used in the analyses. Coloured areas represent ice-free land; different colours denote the ACBRs (see [27]).

**Figure 2:**
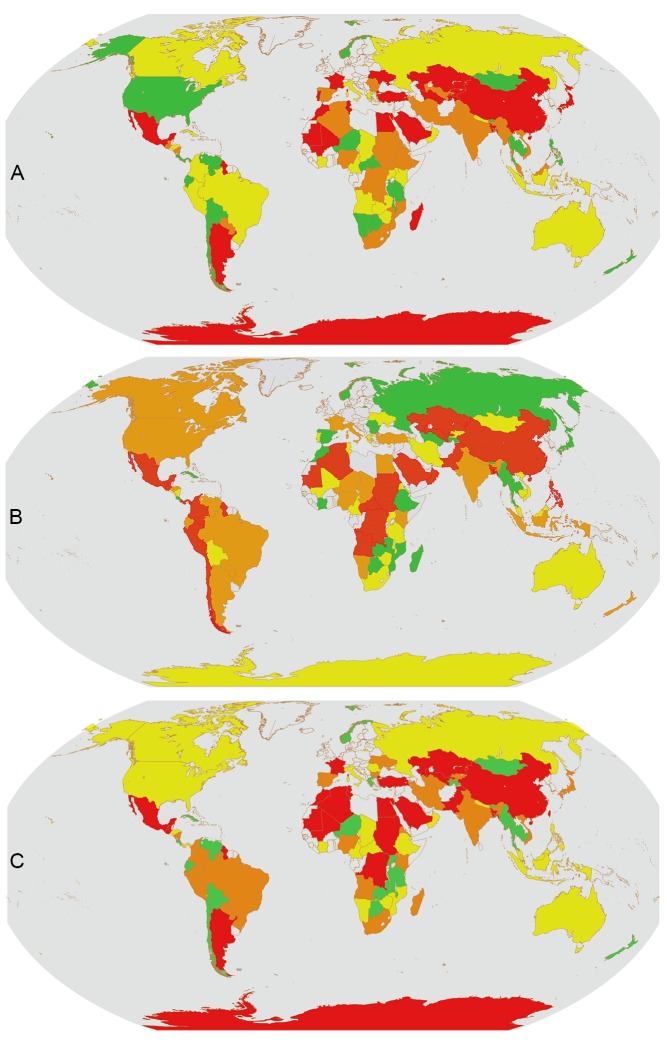
Three measures of protected-area coverage of 83 countries and Antarctica. (A) Total percentage of land protected, (B) protection equality (adapted Gini-coefficient), and (C) integrated protection (the product of A and B). Countries are classified into different coloured quartiles: green  =  highest quartile, yellow  =  second highest quartile, orange  =  second lowest quartile, and red  =  lowest quartile.

Antarctica's ice-free area is 46,253 km^2^, of which only 1.5% is formally designated as a protected area for the purposes of terrestrial biodiversity conservation (688 km^2^). Fifty-five ASPAs have been designated in ice-free areas for their biodiversity values (Figure 1; Tables S1, S2), while a further 18 ASPAs (not considered here) conserve other values, such as historic sites or geologically important features, that are of concern to the ATS [5]. The mean protected area of each Antarctic Conservation Biogeographic Region (ACBR), the equivalent of ecoregions elsewhere (Figure 1) [27], is 1.1%, and no ACBR has 10% or more of its area designated as protected area (range: 0% to 6%; Table S3). In a global context, on a country-comparison basis, Antarctica lies in the lowest quartile for total percentage protection (Figure 2A), mean protected area of each ecoregion, and number of ecoregions with 10% of protection (Figure S1). By any measure, including recently agreed-upon Aichi Target 11, this level of protection is inadequate. While Antarctica ranks in the second-highest quartile for protection equality (Figure 2B) (i.e., the adapted Gini coefficient of Barr et al. [26]), detailed examination of ecoregional protection reveals a less optimistic situation. Five of the fifteen Antarctic ecoregions are not represented in the current portfolio of ASPAs designated for the protection of biodiversity (Figure 1) and two contain most of the protected areas (17 and 10, respectively), representing 74% (503 km^2^) of all ice-free ASPAs designated for the protection of biodiversity. Combining total percentage protection with a protection equality metric, as previously recommended but not implemented globally [26], provides an integrated protection metric by which Antarctica is ranked in the lowest quartile of countries large enough to assess, placed 69th (out of 84), between Mali and Kazakhstan (Figure 2C).

## Protected Areas at Risk of Invasion

**Figure 3:**
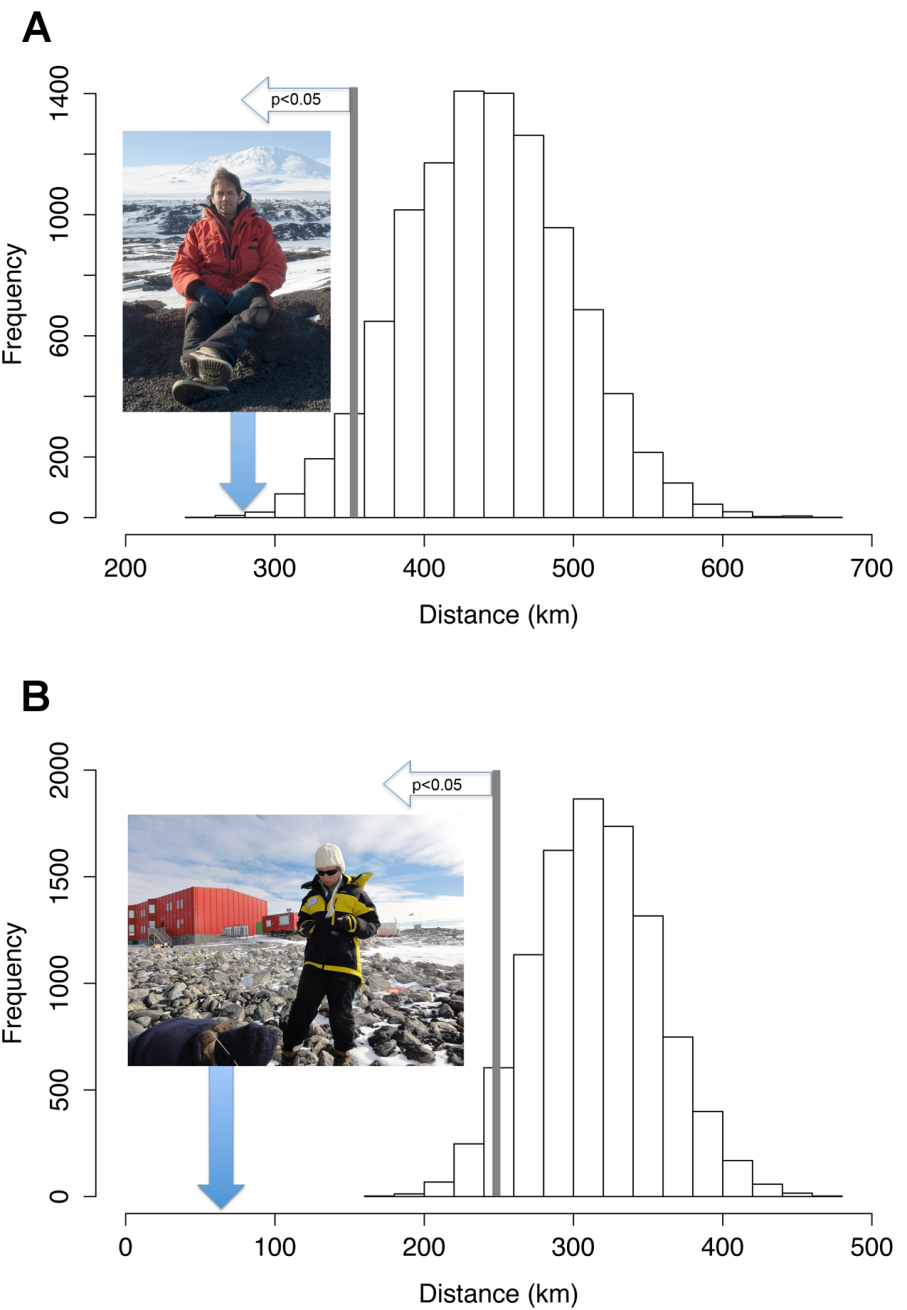
Graphical comparisons of bootstrapped data (histogram) and actual mean distances (image with blue arrow). (A) Distance of ASPAs to tourist landings. Image credit: Aleks Terauds. (B) Distance of ASPAs to landings associated with national programs. Image credit: Dana Bergstrom. Histograms show frequency distribution of 10,000 by means of 55 locations sampled from 1,000 randomly generated, spatial, ice-free locations. Mean distances to ASPAs for both tourists and scientists are well outside the fifth percentile of the histogram (p<0.05), indicating that visitor landings are significantly closer to ASPAs than would be expected by chance.

In terms of risk, the mean distances of ASPAs to tourist landing sites and scientific activity (i.e., established scientific facilities) are 289 km (range: 0 km to 2406 km) and 64 km (range: 0 km to 832 km), respectively, significantly closer than expected for the same number of randomly selected ice-free sites (Figure 3). Seven of the 55 ASPAs, all of which are on the Antarctic Peninsula, are at high risk of nonindigenous species establishment (Figure S2) (risk exceeds 50%, according to Chown et al. [25]), overlapping with high-risk areas for nonindigenous species establishment identified previously [25]. Overall, the mean risk index of establishment of nonindigenous species for ASPAs is 12% (standard error ±5%), significantly higher (by 24 times) than the mean risk for a randomly selected set of ice-free locations (0.5% ±0.1%, χ^2^ = 86.1, p<0.0001).

## A Natural Reserve, Devoted to Peace and Science?

In a global context, the designation of Antarctica as “*a natural reserve, devoted to peace and science*” under the ATS is unique; no other continent has a similar level of apparent protection [6]. This situation may be at least partly responsible for Antarctica's repeated exclusion from global assessments of protected-area effectiveness [26],[28],[29]. However, its apparent protection status reflects management intent, not management outcome [30]. Although the Antarctic environment is less utilised and populated than others, activities permitted on the continent (e.g., road and building construction, vehicle traffic, waste disposal) are having substantial impacts on biodiversity [12],[18],[19].

Aichi Target 11 of the Strategic Plan for Biodiversity 2011–2020 [23] aims for “*at least 17% of terrestrial and inland water areas*” to be protected to ensure conservation of biodiversity. Globally, 13% of terrestrial areas are protected [31]. By comparison, only 1.5% of ice-free terrestrial Antarctica (0.005% of the total continental area) is formally protected for the purposes of biodiversity conservation. Aichi Target 11 also calls for the global protected-area network to be ecologically representative. Again, Antarctica fails to meet this benchmark.

In addition to representing the biodiversity of a given region, protected areas should also safeguard biodiversity from threatening processes [32]. In Antarctica, however, protected areas are significantly closer to sites of human activity than would be expected by chance. This is partly a product of the history of protected-area designation. Such proximity elevates threat, given that human population density adjacent to protected areas is the most significant predictor of their invasive species richness, both in the broader Antarctic region [33] and elsewhere [34]. Moreover, two of the ASPAs at high risk of invasion already support nonindigneous species [14],[35].

## The Way Forward

Antarctic terrestrial biodiversity is concentrated in the continent's relatively small and fragmented ice-free areas. Increasing human activity and risk to biodiversity is also concentrated in these areas, and the total area accorded the additional protection conferred by ASPA status is small. Of the 73 ASPAs, only 55 (688 km^2^) occur in ice-free areas and have recognised terrestrial biodiversity values. In consequence, while there is a widespread general perception that Antarctica is well conserved, in practice conservation of terrestrial biodiversity from a continent-wide perspective is poorly served by the protected-area system. Therefore, what is required now is a systematic network designed to best conserve the biodiversity of Antarctica as a whole. Once a protected area is designated and human activity restricted, management efforts are relatively minimal compared to protected-area management requirements globally [36]. Parties to the Convention on Biological Diversity (CBD) have agreed to improve global protection of biodiversity by encouraging nations to meet the Aichi Targets by 2020. Although Antarctica is excluded from the provisions of the CBD, we believe that the Aichi Targets should be met for the region. For a continent that is so little impacted by human activity compared with the rest of the planet, achieving an objective that has already been attained by several nations should be straightforward for those who manage the region under the Antarctic Treaty System.

## Acknowledgments

We are grateful for constructive comments from P. Convey (British Antarctic Survey), R. Fuller (University of Queensland), and M. A. McGeoch (Monash University). Data utilised in these analyses are contained in the Supporting Information, or the sources referenced therein.

## Supporting Information

Figure S1 **Two measures of protected-area coverage of 83 countries and Antarctica.** (A) Mean percentage protection of ecoregions and (B) percentage of ecoregions with at least 10% protection. We divided the scores of all countries into quartiles for each measure and assigned colours to each quartile: green  =  highest quartile, yellow  =  second highest quartile, orange  =  second lowest quartile, and red  =  lowest quartile.
(PDF)

Figure S2 **Continent-wide risk of establishment of nonindigenous species and high-risk ASPAs.** (See Chown et al. [25] for details of risk index). Inset shows location of ASPAs overlaid on risk index cells with values >0 for the Antarctic Peninsula region.
(PDF)

Table S1 **Designation of ASPAs and their invasion risk.** Designations from Antarctic Protected Areas Database, Secretariat of the Antarctic Treaty, http://www.ats.aq/devPH/apa/ep_protected_detail.aspx?type=2&id=69&lang=e.
(DOCX)

Table S2 **Ice-free ASPAs that have designations related to the protection of terrestrial biodiversity.**
(DOCX)

Table S3 **Overlap of ice-free, biodiversity-designated ASPAs and ACBRs.**
(DOCX)

Methods S1 **Supporting methods.**
(DOCX)

## Abbreviations

ACBR: Antarctic Conservation Biogeographic Region
ASPA: Antarctic Specially Protected Area
ATS: Antarctic Treaty System
CBD: Convention on Biological Diversity
