# Supplementary material for: Antarctica’s Protected Areas Are Inadequate, Unrepresentative, and at Risk
Source: PLoS Biol. 2014 Jun 17;12(6):e1001888. doi: 10.1371/journal.pbio.1001888 (PMC4060989; doi:10.1371/journal.pbio.1001888)

**Figure S1. Two measures of protected-area coverage of 83 countries and Antarctica.** (A) Mean percentage protection of ecoregions and (B) percentage of ecoregions with at least 10% protection. We divided the scores of all countries into quartiles for each measure and assigned colours to each quartile: green = highest quartile, yellow = second highest quartile, orange = second lowest quartile, and red = lowest quartile.

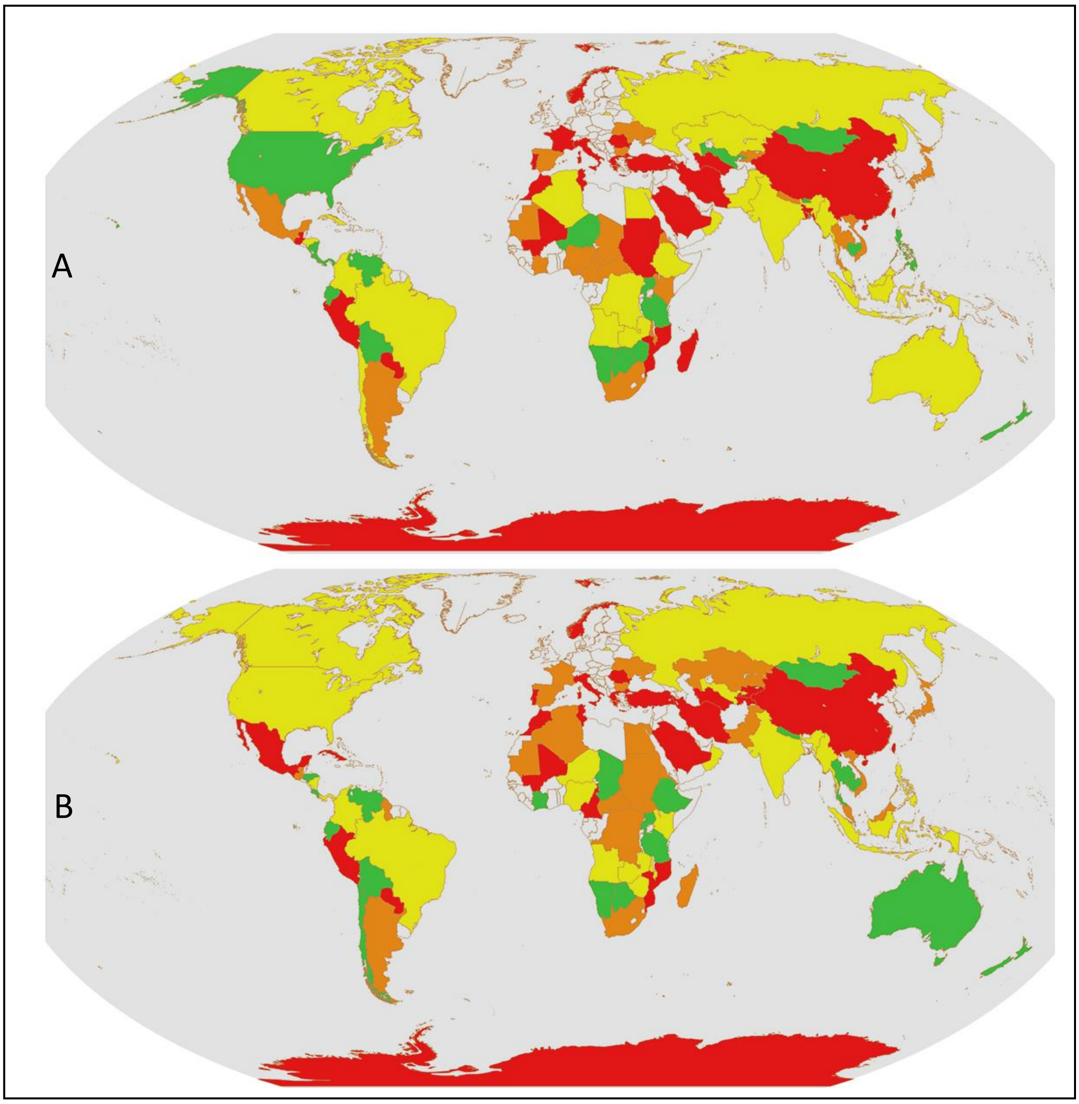

Supplement: Figure S1 — Two measures of protected-area coverage of 83 countries and Antarctica. (A) Mean percentage protection of ecoregions and (B) percentage of ecoregions with at least 10% protection. We divided the scores of all countries into quartiles for each measure and assigned colours to each quartile: green = highest quartile, yellow = second highest quartile, orange = second lowest quartile, and red = lowest quartile. (PDF) [file pbio.1001888.s001.pdf]
