# Supplementary material for: Antarctica’s Protected Areas Are Inadequate, Unrepresentative, and at Risk
Source: PLoS Biol. 2014 Jun 17;12(6):e1001888. doi: 10.1371/journal.pbio.1001888 (PMC4060989; doi:10.1371/journal.pbio.1001888)

Figure S2. Continent-wide risk of establishment of nonindigenous species and high-risk ASPAs. (See Chown et al. [25] for details of risk index). Inset shows location of ASPAs overlaid on risk index cells with values >0 for the Antarctic Peninsula region.

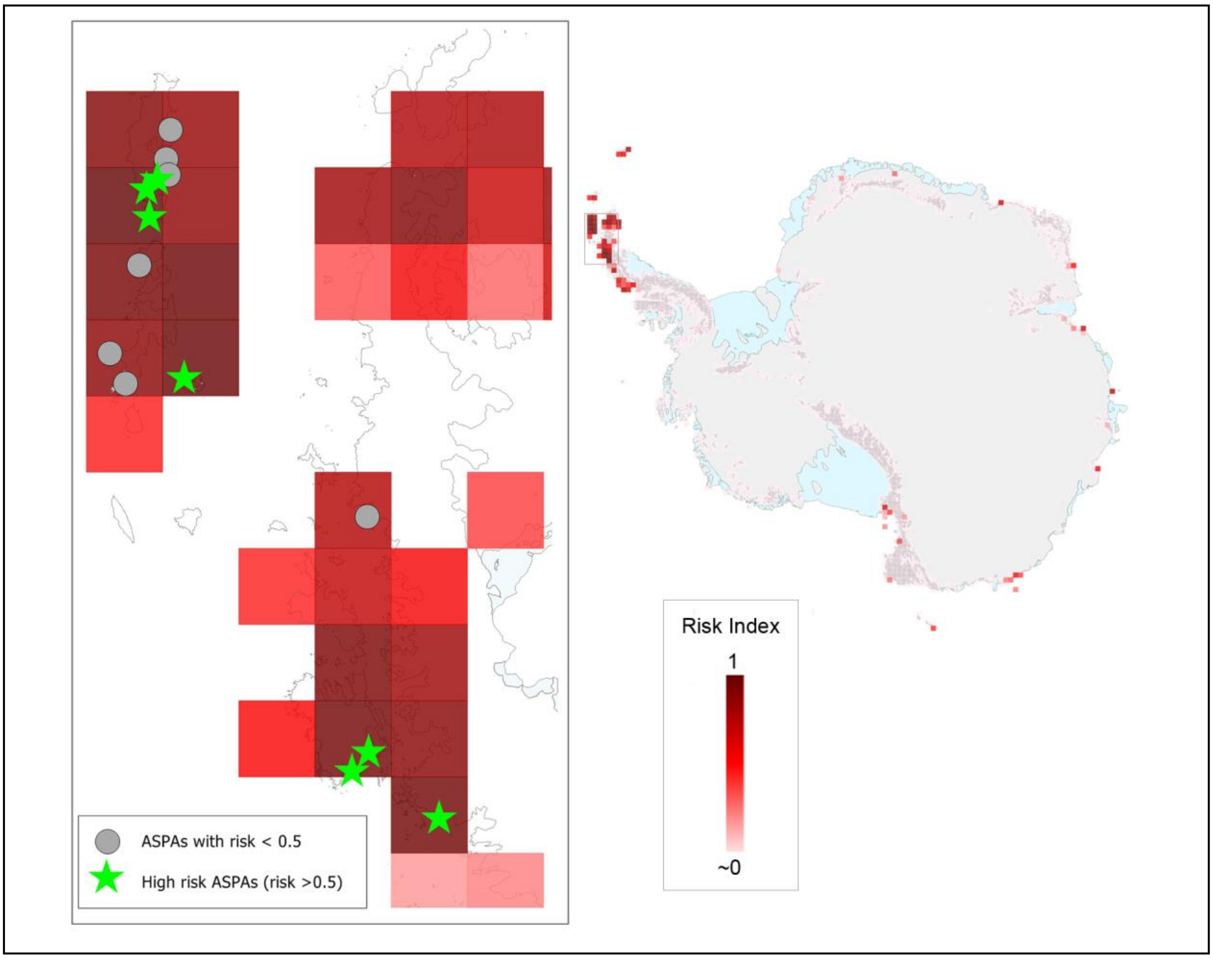

Supplement: Figure S2 — Continent-wide risk of establishment of nonindigenous species and high-risk ASPAs. (See Chown et al. [25] for details of risk index). Inset shows location of ASPAs overlaid on risk index cells with values >0 for the Antarctic Peninsula region. (PDF) [file pbio.1001888.s002.pdf]
