# Supplementary material for: Antarctica’s Protected Areas Are Inadequate, Unrepresentative, and at Risk
Source: PLoS Biol. 2014 Jun 17;12(6):e1001888. doi: 10.1371/journal.pbio.1001888 (PMC4060989; doi:10.1371/journal.pbio.1001888)
Supplement: Table S1 — Designation of ASPAs and their invasion risk. Designations from Antarctic Protected Areas Database, Secretariat of the Antarctic Treaty, http://www.ats.aq/devPH/apa/ep_protected_detail.aspx?type=2&id=69&lang=e. (DOCX) [file pbio.1001888.s003.docx]

**Table S1. Designation of ASPAs and their invasion risk.** Designations from Antarctic Protected Areas Database, Secretariat of the Antarctic Treaty, <http://www.ats.aq/devPH/apa/ep_protected_detail.aspx?type=2&id=69&lang=e>.

| Classification | Reason for designation | Antarctic Specially Protected Areas | Ice-free, biodiversity designated ASPAs at high risk of invasion |
| --- | --- | --- | --- |
| A | Inviolate areas | 2 |  |
| B | Representative of major ecosystems | 10 |  |
| C | Important or unusual assemblage of species | 37 | 6 |
| D | Type locality of known species | 0 |  |
| E | Area of interest to science | 10 | 1 |
| F | Outstanding geological, glaciological geomorphological feature | 5 |  |
| G | Outstanding aesthetic or wilderness value | 1 |  |
| H | Sites or monuments of historic value | 6 |  |
| I | Outstanding environmental, scientific, historic, aesthetic or wilderness values, any combination of those values or on-going planned scientific research | 2 |  |
| Total |  | 73 | 7 |
