# Supplementary material for: Antarctica’s Protected Areas Are Inadequate, Unrepresentative, and at Risk
Source: PLoS Biol. 2014 Jun 17;12(6):e1001888. doi: 10.1371/journal.pbio.1001888 (PMC4060989; doi:10.1371/journal.pbio.1001888)
Supplement: Table S2 — Ice-free ASPAs that have designations related to the protection of terrestrial biodiversity. (DOCX) [file pbio.1001888.s004.docx]

**Table S2.** **Ice-free ASPAs that have designations related to the protection of terrestrial biodiversity.**

| ASPAID | Name | Ice-free area (km) | Latitude | Longitude | Proponent | Original designation | Original designation year | Designation | Modified designation | ACBR ID |
| --- | --- | --- | --- | --- | --- | --- | --- | --- | --- | --- |
| 101 | Taylor Rookery, Mac. Robertson Land | 0.264136 | -67.4548 | 60.8854 | Australia | SPA 1 | 1966 | Rec. IV-1 | Rec. XVII-2 (1992)*, Measure 2 (2005), Measure 1 (2010) | na |
| 102 | Rookery Islands, Holme Bay, Mac. Robertson Land | 1.668916 | -67.5995 | 62.5221 | Australia | SPA 2 | 1966 | Rec. IV-2 | Rec. XVII-2 (1992)*, Measure 2 (2005), Measure 2 (2010) | na |
| 103 | Ardery Island, Budd Coast | 0.54301 | -66.3709 | 110.4528 | Australia | SPA 3 | 1966 | Rec. IV-3 | Rec. XVII-2 (1992)*, Measure 2 (2005), Measure 3 (2010) | 7 |
| 104 | Sabrina Island, Northern Ross Sea, Antarctica | 1.504379 | -66.8997 | 163.317 | New Zealand | SPA 4 | 1966 | Rec. IV-4 | Measure 3 (2009) | na |
| 105 | Beaufort Island | 14.161345 | -76.9469 | 166.9431 | New Zealand | SPA 5 | 1966 | Rec. IV-5 | Measure 1 (1997)*, Measure 2 (2003), Measure 4 (2010) | 9 |
| 106 | Cape Hallett | 0.532818 | -72.3206 | 170.233 | USA | SPA 7 | 1966 | Rec. IV-7 | Rex. XIII-13 (1985), Measure 1 (2002)*, Measure 5 (2010) | 8 |
| 107 | Dion Islands, Marguerite Bay | 4.674938 | -67.8643 | -68.7087 | UK | SPA 8 | 1966 | Rec. IV-8 | Rec. XVI-6 (1991)*, Measure 1 (2002) |  |
| 108 | Green Island, Berthelot Islands | 0.166465 | -65.324 | -64.1509 | UK | SPA 9 | 1966 | Rec. IV-9 | Rec. XVI-6 (1991)*, Measure 1 (2002) | 3 |
| 109 | Moe Island, South Orkney Islands | 1.201153 | -60.7362 | -45.6843 | UK | SPA 13 | 1966 | Rec. IV-13 | Rec. XVI-6 (1991)*, Measure 1 (1995), Measure 1 (2007) | 2 |
| 110 | Lynch Island, South Orkney Islands | 0.14904 | -60.6549 | -45.6084 | UK | SPA 14 | 1966 | Rec. IV-14 | Rec. XVI-6 (1991)*, Measure 1 (2000) | 2 |
| 111 | Southern Powell Island and adjacent islands, South Orkney Islands | 23.556574 | -60.7296 | -45.0053 | UK | SPA 15 | 1966 | Rec. IV-15 | Rec. XVI-6 (1991)*, Measure 1 (1995) | 2 |
| 112 | Coppermine Peninsula, Robert Island | 0.666146 | -62.3741 | -59.712 | Chile | SPA 16 | 1970 | Rec. VI-10 | Rec. XVI-6 (1991)* | 3 |
| 113 | Litchfield Island, Arthur Harbour, Anvers Island | 0.360454 | -64.7704 | -64.0921 | USA | SPA 17 | 1975 | Rec. VIII-1 | Measure 2 (2004)*, Measure 4 (2009) | 3 |
| 114 | Northern Coronation Island, South Orkney Islands | 91.756482 | -60.5618 | -45.5929 | UK | SPA 18 | 1985 | Rec. XIII-10 | Rec. XVI-6 (1991)*, Measure 2 (2003) | 2 |
| 115 | Lagotellerie Island, Marguerite Bay, Antarctic Peninsula | 1.61535 | -67.8869 | -67.404 | UK | SPA 19 | 1985 | Rec. XIII-11 | Rec. XVI-6 (1991)*, Measure 1 (2000) | 3 |
| 116 | New College Valley, Caughley Beach, Cape Bird, Ross Island | 0.337902 | -77.2227 | 166.4422 | New Zealand | SSSI 10, SPA 20 | 1985 | Rec. XIII-8, Rec. XIII-12 | Rec. XVI-7 (1991) E, Rec. XVII-2 (1992)*, Measure 1 (2000), Measure 1 (2006). Measure 1 (2011) | 9 |
| 117 | Avian Island, off Adelaide Island, Antarctic Peninsula | 1.121962 | -67.7727 | -68.8887 | UK | SSSI 30, SPA 21 | 1989 | Rec. XV-6 | Rec. XVI-4, Measure 1 (2002) | 3 |
| 118 | Summit of Mount Melbourne, Victoria Land | 6.56091 | -74.3542 | 164.6845 | New Zealand | SSSI 24, SPA 22 | 1987 | Rec. XIV-5 | Rec. XVI-8*, Measure 2 (2003) 1 | 8 |
| 119 | Davis Valley and Forlidas Pond, Dufek Massif | 56.805772 | -82.488 | -51.1204 | USA | SPA 23 | 1991 | Rec. XVI-9 | Measure 2 (2005), Measure 6 (2010) | 10 |
| 120 | Pointe-Géologie Archipelego, Terre Adélie | 0.08107 | -66.6733 | 140.012 | France | SPA 24 | 1995 | Measure 3 | Measure 2 (2005), Measure 2 (2011) | 13 |
| 121 | Cape Royds, Ross Island | 0.62161 | -77.5564 | 166.16 | USA | SSSI 1 | 1975 | Rec. VIII-4 | Rec. X-6 (1979) E, Rec. XII-5 (1983) E, Rec. XIII-9 (1985), Res. 7 (1995) E, Decision 4 (1998)1, Measure 2 (2000) E, Measure 1 (2002), Measure 5 (2009) | 9 |
| 123 | Barwick Valley, Victoria Land | 418.14355 | -77.3481 | 160.9485 | USA | SSSI 3 | 1975 | Rec. VIII-4 | Rec. X-6 (1979) E, Rec. XII-5 (1983) E, Rec. XIII-7 (1985) E, Res. 7 (1995) E, Measure 2 (2000) E, Measure 1 (2002), Measure 6 (2008) | 9 |
| 124 | Cape Crozier, Ross Island | 72.209636 | -77.4809 | 169.3313 | USA | SSSI 4, SPA 6 | 1966 | Rec. IV-6 | Rec. VIII-4 (1975)*, Rec. VIII-2 (1975), Rec. X-6 (1979) E, Rec. XII-5 (1983) E, Rec. XIII-7 (1985) E, Rec. XVI-7 (1991) E, Measure 3 (2001) E, Measure 1 (2002), Measure 7 (2008) | 9 |
| 126 | Byers Peninsula, Livingston Island | 90.342203 | -62.6321 | -61.0348 | Chile, UK | SSSI 6, SPA 10 | 1966 | Rec. IV-10 | Rec. VIII-4 (1975)*, Rec. VIII-2 (1975), Rec. X-6 (1979) E, Rec. XII-5 (1983) E, Rec. XIII-7 (1985) E, Rec. XVI-5 (1991), Measure 3 (2001) E, Measure 1 (2002), Measure 4 (2011) | 3 |
| 127 | Haswell Island | 5.013384 | -66.5289 | 93.0252 | Russia | SSSI 7 | 1975 | Rec. VIII-4 | Rec. X-6 (1979) E, Rec. XII-5 (1983) E, Rec. XIII-7 (1985) E, Rec. XVI-7 (1991) E, Measure 3 (2001) E, Measure 4 (2005) E, Measure 1 (2006), Measure 5 (2011) | 7 |
| 128 | Western shore of Admiralty Bay | 18.043812 | -62.1976 | -58.4634 | Poland | SSSI 8 | 1979 | Rec. X-5 | Rec. XII-5 (1983) E, Rec. XIII-7 (1985) E, Res. 7 (1995) E, Measure 1 (2000) | 3 |
| 129 | Rothera Point, Adelaide Island | 0.037361 | -67.567 | -68.1157 | UK | SSSI 9 | 1985 | Rec. XIII-8 | Res. 7 (1995) E, Measure 1 (1996), Measure 1 (2007) | 3 |
| 130 | Tramway Ridge, Mount Erebus, Ross Island | 0.04016 | -77.5181 | 167.11 | New Zealand | SSSI 11 | 1985 | Rec. XIII-8 | Rec. XVI-7 (1991) E, Measure 2 (1995), Measure 3 (1997), Measure 1 (2002) | 9 |
| 131 | Canada Glacier, Lake Fryxell, Taylor Valley, Victoria Land | 1.513958 | -77.6115 | 163.0425 | New Zealand | SSSI 12 | 1985 | Rec. XIII-8 | Rec. XVI-7 (1991) E, Measure 3 (1997), Measure 1 (2006), Measure 6 (2011) | 9 |
| 132 | Potter Peninsula | 2.174187 | -62.2562 | -58.609 | Argentina | SSSI 13 | 1985 | Rec. XIII-8 | Res. 3 (1996) E, Measure 3 (1997), Measure 2 (2005) | 3 |
| 133 | Harmony Cove, Nelson Island | 30.689739 | -62.3051 | -59.1946 | Argentina, Chile | SSSI 14 | 1985 | Rec. XIII-8 | Res. 7 (1995) E, Measure 3 (1997), Measure 2 (2005) | 3 |
| 134 | Cierva Point and offshore islands, Danco Coast | 59.025732 | -64.171 | -61.0277 | Argentina | SSSI 15 | 1985 | Rec. XIII-8 | Res. 7 (1995) E Measure 3 (1997), Measure 1 (2006) | 3 |
| 135 | Northeast Bailey Peninsula | 0.281126 | -66.2838 | 110.5443 | Australia | SSSI 16 | 1985 | Rec. XIII-8 | Res. 7 (1995) E, Measure 2 (2000) E, Measure 2 (2003), Measure 8 (2008) | 7 |
| 136 | Clark Peninsula | 9.380319 | -66.251 | 110.5967 | Australia | SSSI 17 | 1985 | Rec. XIII-8 | Res. 7 (1995) E Measure 1 (2000), Measure 1 (2006), Measure 7 (2009) | 7 |
| 137 | North-West White Island, McMurdo Sound | 141.60863 | -78.0416 | 167.3051 | USA | SSSI 18 | 1985 | Rec. XIII-8 | Rec. XVI-7 (1991) E, Measure 3 (2001) Ç, Measure 1 (2002), Measure 9 (2008) | 9 |
| 138 | Linnaeus Terrace, Asgard Range, Victoria Land | 0.782374 | -77.5978 | 161.0826 | USA | SSSI 19 | 1985 | Rec. XIII-8 | Res. 7 (1995) E, Measure 1 (1996), Measure 10 (2008) | 9 |
| 139 | Biscoe Point, Anvers Island | 0.094287 | -64.806 | -63.7753 | USA | SSSI 20 | 1985 | Rec. XIII-8 | Res. 3 (1996) Ç, Decision 4 (1998)1, Measure 2 (2000) E, Measure 2 (2004), Measure 7 (2010) | 3 |
| 140 | Parts of Deception Island | 1.009377 | -62.9207 | -60.6691 | UK | SSSI 21 | 1985 | Rec. XIII-8 | Res. 7 (1995) E, Measure 2 (2000) E, Measure 3 (2005) | 3 |
| 141 | Yukidori Valley Langhovde, Lützow-Holmbukta | 4.876129 | -69.2373 | 39.7749 | Japan | SSSI 22 | 1987 | Rec. XIV-5 | Rec. XVI-7 (1991) E, Measure 1 (2000) | 5 |
| 142 | Svarthamaren, Mühlig-Hofmannfjella, Dronning Maud Land | 6.491545 | -71.9089 | 5.1825 | Norway | SSSI 23 | 1987 | Rec. XIV-5 | Res. 3 (1996) E, Measure 1 (1999), Measure 2 (2004), Measure 8 (2009) | 6 |
| 143 | Marine Plain, Mule Peninsula, Vestfold Hills, Princess Elizabeth Land | 20.462391 | -68.6308 | 78.1419 | Australia | SSSI 25 | 1987 | Rec. XIV-5 | Res. 3 (1996) E, Measure 2 (2000) E, Measure 2 (2003) | 7 |
| 147 | Ablation Point, Ganymede Heights, Alexander Island | 109.02115 | -70.8524 | -68.4153 | UK | SSSI 29 | 1989 | Rec. XV-6 | Res. 3 (1996) E, Measure 2 (2000) E, Measure 1 (2002) | 4 |
| 149 | Cape Shirreff, Livingston Island | 9.736266 | -62.4747 | -60.8034 | USA | SSSI 32, SPA 11 | 1966 | Rec. IV-11 | Rec. XV-7 (1989)*, Res. 3 (1996) E, Decision 4 (1998)1, Measure 2 (2000) E, Measure 2 (2005), Measure 7 (2011) | 3 |
| 150 | Ardley Island, Maxwell Bay, King George Island | 1.21698 | -62.212 | -58.9315 | Chile | SSSI 33 | 1991 | Rec. XVI-2 | Measure 3 (2001) E, Measure 4 (2005) E, Measure 9 (2009) | 3 |
| 151 | Lions Rump | 1.322779 | -62.1342 | -58.1379 | Poland | SSSI 34 | 1991 | Rec. XVI-2 | Decision 4 (1998)1, Measure 1 (2000) | 3 |
| 154 | Botany Bay, Cape Geology, Victoria Land | 2.139478 | -77.0106 | 162.5678 | New Zealand | SSSI 37 | 1997 | Measure 3 | Measure 2 (2003), Measure 11 (2008) | 9 |
| 160 | Frazier Islands | 0.064759 | -66.225 | 110.1535 | Australia |  | 2003 | Measure 2 | Measure 13 (2008) | 7 |
| 163 | Dakshin Gangotri Glacier, Dronning Maud Land | 4.310395 | -70.7474 | 11.582 | India |  | 2005 | Measure 2 | Measure 12 (2010) | 6 |
| 164 | Scullin and Murray Monoliths, Mac Robertson Land, East Antarctica | 10.2 | -67.7922 | 66.7111 |  |  |  |  |  | 8 |
| 165 | Edmonson Point, Wood Bay, Victoria Land | 5.497826 | -74.3255 | 165.0956 | Italy |  | 2006 | Measure 1 | Measure 8 (2011) | 8 |
| 167 | Hawker Island | 2.174501 | -68.6355 | 77.8578 | Australia |  | 2006 | Measure 1 | Measure 9 (2011) | 7 |
| 169 | Amanda Bay, Ingrid Christensen Coast, Princess Elizabeth Land | 17.146826 | -69.2536 | 76.8475 | Australia, China |  | 2008 | Measure 3 |  | 7 |
| 170 | Marion Nunataks, Charcot Island, Antarctic Peninsula | 179.55357 | -69.7583 | -75.25 | UK |  | 2008 | Measure 4 |  | 4 |
| 171 | Narebski Point, Barton Peninsula, KGI | 0.891893 | -62.2339 | -58.7679 | Korea |  | 2009 | Measure 13 |  | 3 |
| 173 | Wellington Point (terrestrial) and Silverfish Bay (marine) | 5.65 | -74.6183 | 164.96 | USA |  | 2013 |  |  | 8 |
