# Supplementary material for: Antarctica’s Protected Areas Are Inadequate, Unrepresentative, and at Risk
Source: PLoS Biol. 2014 Jun 17;12(6):e1001888. doi: 10.1371/journal.pbio.1001888 (PMC4060989; doi:10.1371/journal.pbio.1001888)
Supplement: Table S3 — Overlap of ice-free, biodiversity-designated ASPAs and ACBRs. (DOCX) [file pbio.1001888.s005.docx]

**Table S3.** **Overlap of ice-free, biodiversity-designated ASPAs and ACBRs.**

| Region ID | ACBR name | Approximate area ACBR (km^2^) | Number ASPAs in ACBR | Area of overlap (km^2^) | % of ACBR covered |
| --- | --- | --- | --- | --- | --- |
| ACBR 1 | North-east Antarctic Peninsula | 1142 | 0 | 0 | 0 |
| ACBR 2 | South Orkney Islands | 148 | 4 | 8.8 | 5.9 |
| ACBR 3 | North-west Antarctic Peninsula | 5081 | 17 | 93.7 | 1.8 |
| ACBR 4 | Central south Antarctic Peninsula | 4959 | 2 | 75.6 | 1.5 |
| ACBR 5 | Enderby Land | 2152 | 1 | 4.85 | 0.2 |
| ACBR 6 | Dronning Maud Land | 5500 | 2 | 9.15 | 0.2 |
| ACBR 7 | East Antarctica | 1085 | 8 | 27.8 | 2.6 |
| ACBR 8 | North Victoria Land | 9522 | 4 | 13.95 | 0.14 |
| ACBR 9 | South Victoria Land | 10368 | 10 | 409 | 3.9 |
| ACBR 10 | Transantarctic Mountains | 19347 | 1 | 44.6 | 0.2 |
| ACBR 11 | Ellsworth Mountains | 2965 | 0 | 0 | 0 |
| ACBR 12 | Marie Byrd Land | 1158 | 0 | 0 | 0 |
| ACBR 13 | Adelie Land | 178 | 1 | 0.05 | 0.03 |
| ACBR 14 | Ellsworth Land | 220 | 0 | 0 | 0 |
| ACBR 15 | South Antarctic Peninsula | 2990 | 0 | 0 | 0 |
