# Supplementary material for: Antarctica’s Protected Areas Are Inadequate, Unrepresentative, and at Risk
Source: PLoS Biol. 2014 Jun 17;12(6):e1001888. doi: 10.1371/journal.pbio.1001888 (PMC4060989; doi:10.1371/journal.pbio.1001888)
Supplement: Methods S1 — Supporting methods. (DOCX) [file pbio.1001888.s006.docx]

**Supporting methods.**

Our assessment was conducted in several steps. We first quantified the amount of protected terrestrial ice-free land and utilised the recently completed work on Antarctic Conservation Biogeographic Regions [27] together with the location and number of ASPAs to calculate protection metrics for the region. The protection metrics were:

a) Total proportion of protected area, b) Mean fraction of each bioregion protected, c) Number of regions that have more than 10% of their area protected, d) The protection equality [26] for the whole of Antarctica, and a new metric, combining a) and d), we call e) Integrated Protection. The latter has previously been suggested [26] but not further developed or implemented.

*Spatial base layers*

To undertake the analyses a number of spatial data layers were required, including areas of exposed rock (hereafter ice-free areas) and ASPA locations and visitor locations, the ice-free area of Antarctica was calculated using the Antarctic Digital Database Version 5 exposed rock shapefile (scale of capture resolution - © Scientific Committee for Antarctic Research 1993-2006) with a Lambert equal-area projection.

Maps showing the locations and extent of Antarctic Specially Protected Areas (points and polygons) were compiled by Environmental Research and Assessment Consultants; and were provided by the Australian Antarctic Division (AAD) (publicly available at <http://data.aad.gov.au/aadc/biodiversity>). We examined all ASPA management plans (available at [www.ats.aq](http://www.ats.aq)) and identified those ice-free APSAs with terrestrial biodiversity values specifically identified in the management plans. They include unique or vulnerable communities and/or an important vascular plant, bryophytes, lichens, and bird colonies. Most of these communities also support invertebrates, although they are rarely mentioned explicitly in management plans. ASPAs in marine areas were excluded, as were those designated for their underlying historical, memorial and/or geological values (Table S1).

We used the recently delineated Antarctic Conservation Biogeographic Regions [27] as the equivalent of Antarctic ecoregions. The spatial layer delineating location and extent of the ACBRs was downloaded from <http://data.aad.gov.au/aadc/biodiversity>. We compiled maps showing global metrics of protection using data from the World Database on Protected Areas (2013 update - <http://www.wdpa.org/>) and Barr et al. [26] and these were used to provide a global context for the Antarctic protection metrics we calculate here.

*Protection Metrics*

To compare the level of protection in Antarctica with the rest of the world, the following metrics were calculated utilising the above spatial layers:

a) Total proportion of protected area = ${\sum_{i=1}^{n} A_{fi}}/A$

Where *A_fi_* = area of each ASPA, indexed by *i*, that overlaps with ice-free areas (calculated by ‘clipping’ the ASPA polygons with the ice free area layer), *n* is the number of ASPAs and *A* is the total ice free area (calculated by summing all ice free polygons in ice-free layer).

b) Mean fraction of each bioregion protected = $\frac{\sum_{1}^{15} \left( {\sum_{1}^{m_{j}} A_{fij}}/{A_{j}} \right)}{15}$

Where *m_j_* is the number of ASPAs in ACBR *j* and *A_fij_* is the area of each ASPA, *i*, overlapping with ice-free areas of ACBR *j*. *A_j_* is the total area of ACBR *y*. Note: *y* =1-15 (15 ACBRs).

c) Number of bioregions that have more than 10% of their total area protected = Number of times that $\left( {\sum_{1}^{m_{j}} A_{fij}}/{A_{j}} \right) >0.1$

d) Protection equality: This protection metric was developed by Barr et al. [26] and is a measure of how equitably the different ecoregions (ACBRs) are represented in the ASPA system.

e) Integrated protection: In a thorough analysis of these metrics, Barr et al. [26] suggested that a more representative metric could be obtained by combining the overall level of protection (a) with the Protection Equality (d). By taking the product of these two measures we calculated integrated protection for 83 countries and continental ice-free Antarctica.

*Proximity of ASPAs to human activity*

Tourist landing data were compiled from information supplied by the International Association of Antarctica Tour Operators (<http://iaato.org/tourism-statistics>) and represented all ice-free tourist landings from 1/7 2007- 30/6/2008. Tourist numbers over this time period represent a peak in visitation, however, are considered representative of current and future numbers [20]. Data on human visits associated with national programs was obtained from data in the Council of Managers of National Antarctic Programs 2010 Annual Report [37] and established scientific facilities on ice-free areas. Chown et al. [25] also quantified the risk of non-native species establishment in Antarctica by taking into account a number of factors to calculate a gridded risk index (ranging from 0 to 100%) for terrestrial ice-free Antarctica. All spatial layer manipulation and analyses were carried out using Manifold Systems Professional GIS (V8.0.28).

The biodiversity designated and ice-free ASPA layer was overlaid on the visitor landing layers and distances measured to the closest tourist or scientific landing point. Risk-index values were also assigned to the corresponding ASPA point location. ASPAs that did not overlap with any cell were assigned a risk index of 0. To test the significance of the visitor proximity and risk index one thousand random ice-free locations were generated and 55 values (to correspond to the number of biodiversity, ice-free ASPAs) were extracted 10 000 times and the mean taken each time. The frequency distribution of these means was then compared to the actual means. This process was undertaken for tourist mean distance, station mean distance and risk index. All of these analyses were implemented using R [38].

*Methods References*

37. COMNAP (2009) COMNAP Report to ATCM XXXII. Appendix 2 Main Antarctic Facilities operated by the National Antarctic Programs in 2009 in the Antarctic Treaty Area. Antarctic Treaty Consultative Meeting XXXII Committee for Environmental Protection XII. Information Paper 105 submitted by Council of Managers of National Antarctic Programs. Baltimore. <http://www.ats.aq/documents/ATCM32/att/ATCM32_att078_e.pdf> . Accessed 17 February 2014.

38. R Core Team (2013) R: A language and environment for statistical computing. R Foundation for Statistical Computing, Vienna, Austria. URL <http://www.R-project.org/> . Accessed 4 January 2014.
